# Performance optimization of a bendable parabolic cylinder collimating X-ray mirror for the ALS micro-XAS beamline 10.3.2

**DOI:** 10.1107/S1600577515001459

**Published:** 2015-04-08

**Authors:** Valeriy V. Yashchuk, Gregory Y. Morrison, Matthew A. Marcus, Edward E. Domning, Daniel J. Merthe, Farhad Salmassi, Brian V. Smith

**Affiliations:** a Advanced Light Source, Lawrence Berkeley National Laboratory, One Cyclotron Road, MS 15R0317, Berkeley, CA 94720, USA; b Engineering Division, Lawrence Berkeley National Laboratory, One Cyclotron Road, MS 15R0317, Berkeley, CA 94720, USA; c Center for X-ray Optics, Lawrence Berkeley National Laboratory, One Cyclotron Road, MS 15R0317, Berkeley, CA 94720, USA

**Keywords:** X-ray mirrors, bendable mirrors, at-wavelength metrology, optimal surface shaping

## Abstract

The Advanced Light Source (ALS) beamline (BL) 10.3.2 is an apparatus for X-ray microprobe spectroscopy and diffraction experiments, operating in the energy range 2.4–17 keV. The performance of the beamline, namely the spatial and energy resolutions of the measurements, depends significantly on the collimation quality of light incident on the monochromator. In the BL 10.3.2 end-station, the synchrotron source is imaged 1:1 onto a set of roll slits which form a virtual source. The light from this source is collimated in the vertical direction by a bendable parabolic cylinder mirror. Details are presented of the mirror design, which allows for precision assembly, alignment and shaping of the mirror, as well as for extending of the mirror operating lifetime by a factor of ∼10. Assembly, mirror optimal shaping and preliminary alignment were performed *ex situ* in the ALS X-ray Optics Laboratory (XROL). Using an original method for optimal *ex situ* characterization and setting of bendable X-ray optics developed at the XROL, a root-mean-square (RMS) residual surface slope error of 0.31 µrad with respect to the desired parabola, and an RMS residual height error of less than 3 nm were achieved. Once in place at the beamline, deviations from the designed optical geometry (*e.g.* due to the tolerances for setting the distance to the virtual source, the grazing incidence angle, the transverse position) and/or mirror shape (*e.g.* due to a heat load deformation) may appear. Due to the errors, on installation the energy spread from the monochromator is typically a few electron-volts. Here, a new technique developed and successfully implemented for at-wavelength (*in situ*) fine optimal tuning of the mirror, enabling us to reduce the collimation-induced energy spread to ∼0.05 eV, is described.

## Abstract 2

Details of an original design of a bendable parabolic cylinder collimating X-ray mirror with active temperature stabilization are presented. The design grants assembly without spurious stresses, optimal alignment and shaping of the mirror. A motorized sagittal motion allows new areas of the mirror to be used when the current area is damaged or contaminated.

## 1. Introduction   

Beamline (BL) 10.3.2 at the Advanced Light Source (ALS) is a versatile environmental and materials science tool, primarily designed for heavy metal speciation and location. With a 1.27 T bending-magnet source, it operates in the energy range 2.45–17 keV. The X-ray beam is focused *via* a Kirkpatrick–Baez (KB) mirror system (Kirkpatrick & Baez, 1948 ▶) to the sample location with a spot size of 1–15 µm. A full description of this beamline and its capabilities, as of 2004, is given by Marcus *et al.* (2004 ▶).

**Figure 1:**
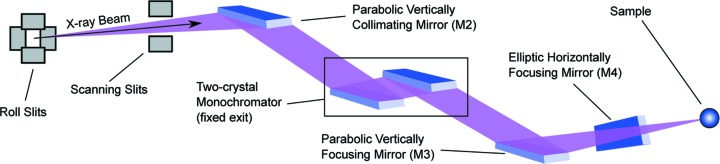
Layout of the ALS beamline 10.3.2 end-station optical system.

The source is imaged by a side-deflecting toroidal mirror with unit magnification onto a pair of roll slits. These slits can be adjusted in size from 0 to 2 mm and serve as the virtual source for the downstream focusing optics, shown schematically in Fig. 1 ▶. Between the roll slits and the parabolic cylinder vertically collimating mirror (M2) are a pair of JJ X-ray^TM^ adjustable scanning slits used for *in situ* metrology. The nominal focal length of M2 measured from its centre to the roll slits is 1333.66 mm. The diverging, ideally spherical, wavefront produced by the roll slits is collimated in the vertical direction by M2 in preparation for transmission through the two-crystal monochromator. A second parabolic cylinder mirror (M3) focuses the beam vertically, and an elliptical mirror (M4) focuses the beam horizontally. Mirrors M2, M3 and M4 are all bendable mirrors, based on a similar design approach (Yuan *et al.*, 2010*a* ▶), each with two bending, cantilever-like, couples attached to ends of the mirror substrate. The image of the roll slits at the sample location has a calculated demagnification of 18.41 and 5.02 in the vertical and horizontal directions, respectively.

The performance of the beamline, namely the spatial and energy resolutions of the measurements, depends significantly on the collimation quality of light incident on the monochromator. In the present paper, we systematically describe the design approaches and experimental *ex situ* and *in situ* techniques developed and successfully implemented for optimization of beamline quality of the M2 bendable parabolic cylinder mirror, which enabled us to significantly improve the operating performance of the ALS microspectroscopy beamline 10.3.2.

This paper is organized as follows. Details of an original design of the new M2 bendable parabolic cylinder collimating mirror for BL 10.3.2 with active temperature stabilization are presented in §2. The design allows assembly without spurious stresses, as well as precision alignment and shaping of the mirror. Due to a motorized sagittal translation, multiple sagittal shifts are possible in order to expose new areas of the working surface and, therefore, for a longer operating lifetime of the mirror. §3 and §4 describe procedures used in the ALS X-ray Optics Laboratory (XROL) for precision assembly, alignment and shaping of the mirror prior to beamline installation. Once in place at the beamline, slight deviations from the design of the optical geometry (*e.g.* due to the tolerances for setting the distance to the virtual source and/or the grazing incidence angle) and/or mirror shape (*e.g.* due to a heat load deformation) can appear. §5 demonstrates an original technique for *in situ* optimal tuning of the M2 collimating mirror using the transmission properties of the monochromator. The paper concludes (§6) by summarizing the main concepts and results discussed throughout the paper.

## 2. M2 mirror assembly   

At the experimental conditions of BL 10.3.2 and without heat dissipation mechanisms, the incident X-ray beam can significantly raise the temperature of the end-station mirrors in vacuum, especially mirror M2. The absorbed heat distorts the mirror shape, which had been precisely tuned *ex situ*. There are also environmental temperature shifts, which can lead to drifts of the mirror shape. In order to address these problems, a new approach to design of bendable X-ray mirrors with active temperature stabilization has been suggested and tested at the ALS XROL (Yuan *et al.*, 2010*a* ▶). The effectiveness of the approach for a number of applications at the ALS has been demonstrated (Kunz *et al.*, 2009 ▶; Yuan *et al.*, 2010*a* ▶; Yashchuk *et al.*, 2013*a* ▶). The construction of the mirrors M2, M3 and M4 of BL 10.3.2 are based on this design.

**Figure 2:**
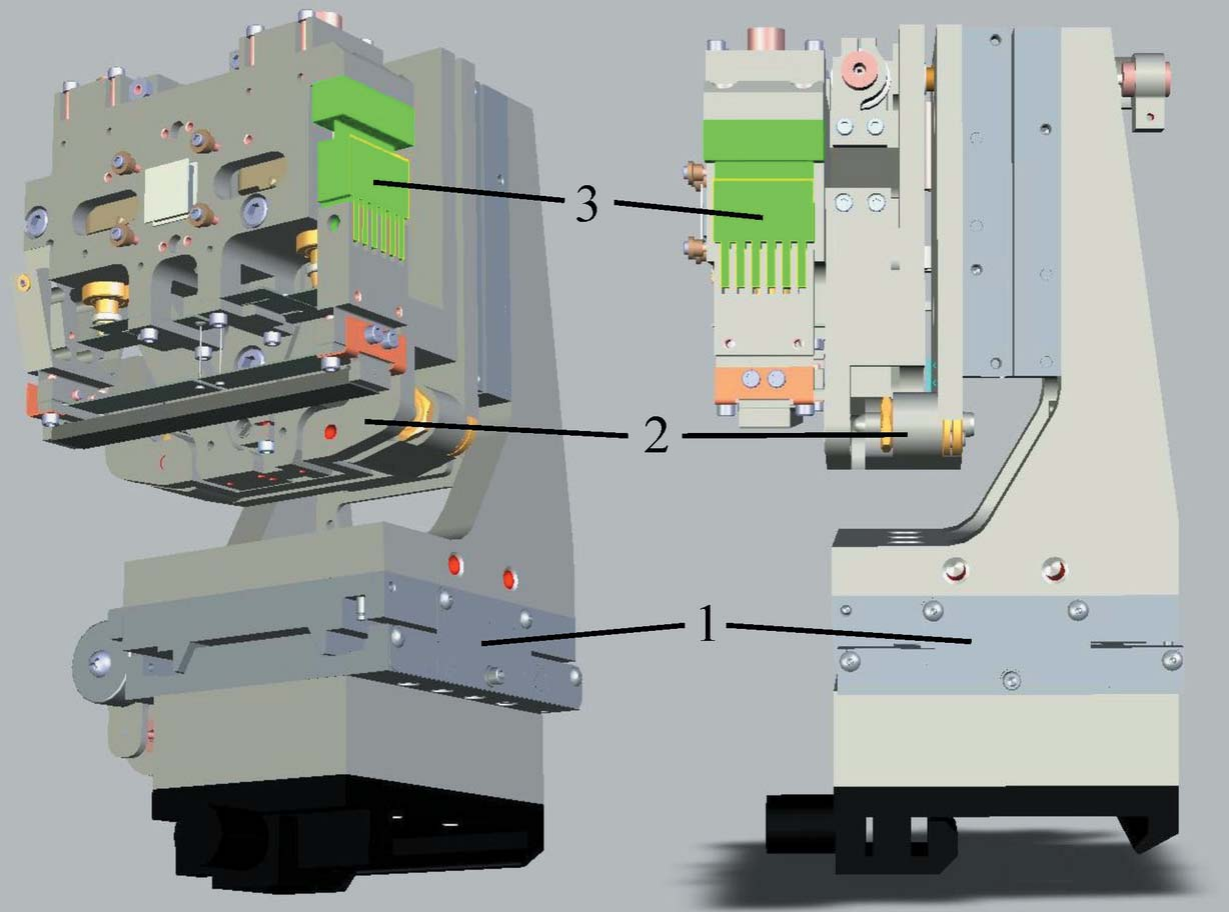
(*a*) Front view and (*b*) side view of the new assembly design with (1) sagittal translation mechanism, (2) roll mechanism with near-surface pivot point and (3) new interleaving hinge joint in post structure.

**Figure 3:**
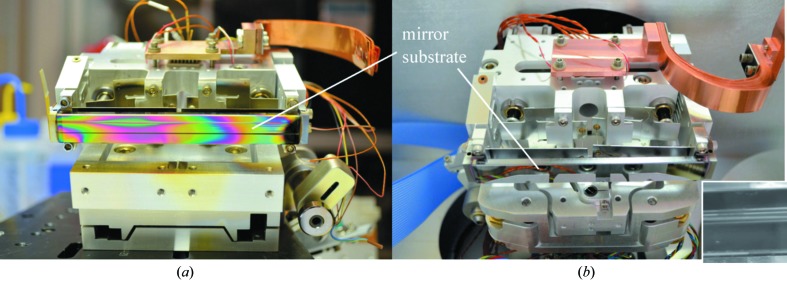
(*a*) Previous mirror assembly of M2 with extensive damage on substrate due to carbon contamination, and (*b*) new mirror assembly described in the text. The inset in (*b*) shows a fragment of the mirror substrate after usage of the mirror with four different sagittal positions. A significant part of the mirror is damaged; however, about six more shifts are still possible.

In the design, the mirror substrate is attached to the assembly with two aluminium posts (Fig. 2 ▶). Molybdenum end-pieces, glued to the substrate (Hartman *et al.*, 1998 ▶), connect the substrate to the posts, in order to better facilitate heat transfer. At room temperature, the thermal expansion of the molybdenum matches well to that of silicon. The thermal conductivity of molybdenum (at room temperature) is approximately 138 W m^−1^ K^−1^, smaller than that of aluminium (∼237 W m^−1^ K^−1^) by a factor of less than two, and larger than that of Invar (∼14 W m^−1^ K^−1^), which is commonly used in similar applications, by a factor of approximately ten. The mirror design and the selection of these materials allow efficient temperature stabilization of the substrate with a Peltier element attached directly to the body of the mirror assembly with an indium foil in-between (Fig. 3 ▶).

Bending of the substrate is achieved with two cantilever springs. Each cantilever spring is connected through a wire to a displacement-reduction spring driven by Picomotor^TM^ actuators (Fig. 2 ▶). The displacement of the actuators is monitored with linear variable differential transformers, LVDTs (Macro Sensors^1^). The bender design allows extremely fine control of the bending couples applied to the mirror substrate. In order to decouple the substrate with the bending mechanism from the mounting posts, the latter have a thin flexure with a neck of thickness 380 µm. The thickness value is a compromise between the requirements to flexibility and thermal conductivity. One post has a twist correction mechanism, designed with its axis of rotation on the reflecting surface of the mirror. The anti-twist adjustment is performed manually in the course of assembly of the mirror.

The base vacuum inside the beamline end-station vacuum chamber is relatively low, about 4 × 10^−5^ Pa. This leads to significant carbon contamination of the optics inside. Fig. 3(*a*) ▶ shows the damaged old substrate of M2, observed upon its removal from the beamline. In order to mitigate this problem, oxygen is flowed through the chamber up to pressures of ∼10^−3^ Pa during user operations. As can be seen, this does not completely resolve the issue. The damage is much more severe along the beam footprint, as clearly seen in Fig. 3(*a*) ▶. For BL 10.3.2 applications, the rest of the mirror surface can still be effectively used if the mirror is slightly shifted in the sagittal direction. This opportunity is exploited in the new design of the M2 collimating mirror.

Fig. 3(*b*) ▶ shows the M2 assembly with a brand new substrate. The super-polished single-crystal silicon substrate [approximately 102 mm (length) × 12 mm (width) × 6 mm (thickness)] was coated at the LBNL Center for X-ray Optics (CXRO), using magnetron sputtering in argon atmosphere. The coating layers consisted, from bottom to top, of a 5 nm-thick adhesive layer of chromium, a 25 nm-thick layer of gold and a 8 nm-thick layer of ruthenium.

Matching at large the mirror design described by Yuan *et al.* (2010*a* ▶), the new mirror assembly, presented here, includes three major new features.

First, a motorized translation stage is added to provide in-vacuum sagittal translation of the mirror. When the carbon contamination renders a portion of the mirror surface unusable, the mirror can be remotely translated sagittally within a ±2.5 mm range to illuminate a different, less damaged, part of the surface. The beam has a sagittal width of about 0.4 mm on the mirror surface, and therefore the operational lifetime of the mirror at the beamline is increased by a factor of ∼10 by applying successive 0.5 mm shifts of the mirror in the sagittal direction.

Second, a motorized roll mechanism with a pivot point located near the mirror surface (Fig. 3*b* ▶) was integrated into the mirror design. The roll mechanism allows for precision mutual roll alignment of the BL 10.3.2 end-station optics. Placing the pivot point close to the mirror surface ensures that *in situ* roll adjustments have a minimal effect on the overall positioning of the mirror. Before installation, the mutual roll alignment of the M2, M3 and M4 mirrors is performed at the XROL (see §3). If necessary, the motorized roll stages of the M2 and M4 mirrors allows *in situ* optimization of the mirrors’ roll orientation with respect to the monochromator.

Third, one of the posts, supporting the substrate, is designed as a flexural (folding) structure with an interleaving hinge joint, highlighted in Fig. 2 ▶ in green. A mismatch of size and/or angle between the substrate and the post upon gluing can lead to a compression pre-stress on the mounted substrate and, therefore, contribute to a large residual curvature with a radius comparable with that of the desired parabolic shape (∼660 m). The thin-neck flexures provide some flexibility in the posts, partially alleviating the stresses caused by such misalignments. The folding post structure completely solves the problem. The interleaving hinge joint provides significantly increased flexibility of the assembly and, therefore, reduces stresses on the substrate, while maintaining the structural integrity of the post when the joint is locked.

The mirror is also equipped with motorized tilting and translation stages used for adjustment of the grazing-incidence angle and the vertical position of the mirror, respectively. All the alignment stages are driven by Picomotor^TM^ actuators, close-looped with dedicated LVDTs. The software for automated control of the stages is developed on the LabView^TM^ platform.

## 3. Mirror assembly, alignment and pre-shaping with a Fizeau interferometer   

The assembly, preliminary alignment and shaping of the mirror are performed by monitoring the mirror surface shape with a six-inch ZYGO^TM^ GPI Fizeau interferometer at the ALS XROL.

First, with relaxed cantilever springs and unlocked interleaving hinge joint, the mirror substrate, with glued end-blocks, is attached to the posts (Fig. 2 ▶). Positioning of the substrate and tightening of the end-pieces screws (two screws on each side) is done in such a way as to provide the smallest possible curvature and twist of the installed substrate. The screws of each pair have opposite handedness; one screw has a right-hand thread, while the other one is left-handed. In the course of assembly, simultaneous tightening of both screws helps reducing position backlash, stress and twist of the substrate. Second, while continuing to observe normal-incidence interferograms of the mirror surface, we lock the interleaving hinge joint of the folding post, ensuring a minimal pre-shape of the substrate.

In the previous design of mirror M2, a mismatch of size and/or angle between the substrate and the mounting posts lead to a pre-bending of the mirror (that is, with zero torque applied to the bending couples) with a radius of curvature of ∼700 m, comparable with the desired one. The curvature was compensated by inelastic bending the thin-neck flexures of the posts. The procedure was found to be very difficult to control. With the new design, by fine adjustment of the flexural post with simultaneous monitoring of the mirror curvature with the interferometer, it is relatively easy to reduce the initial pre-bending of the substrate so that the radius of curvature exceeds 7 km.

**Figure 4:**
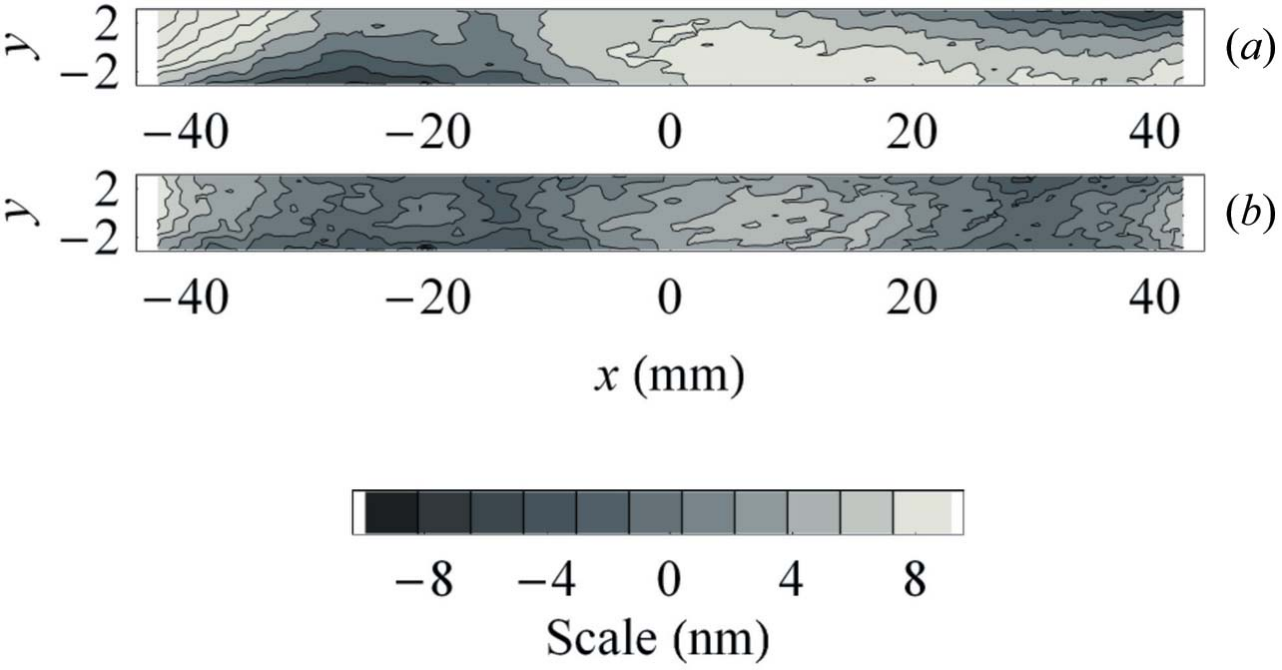
Height error measured with the interferometer, before (*a*) and after correcting (*b*) for twist error.

Next, the twist in the mirror substrate is removed. The twist appears due to the finite accuracy of placement of the glued end pieces and that of mounting of the substrate to the mirror assembly. Fig. 4(*a*) ▶ shows the residual surface height error, after subtracting the best-fit cylindrical shape from the mirror height map, measured with the interferometer. The twist error is clearly seen as reversed sagittal trends of surface height at the left and the right ends of the substrate with peak-to-valley variation of about 20 nm. The twist error was almost totally compensated by adjusting the twist correction mechanism of the mirror. The resulted residual height error is almost random, with the RMS variation of about 3 nm (Fig. 4*b* ▶).

Finally, measuring the surface figure with the interferometer, the mirror is bent to a shape close to the desired cylindrical parabola and the anti-twist correction is applied one more time (if necessary). Later, a final, more precise, anti-twist correction is performed using a surface-slope-measuring long trace profiler (LTP) after precise tuning of the mirror surface shape (§4).

**Figure 5:**
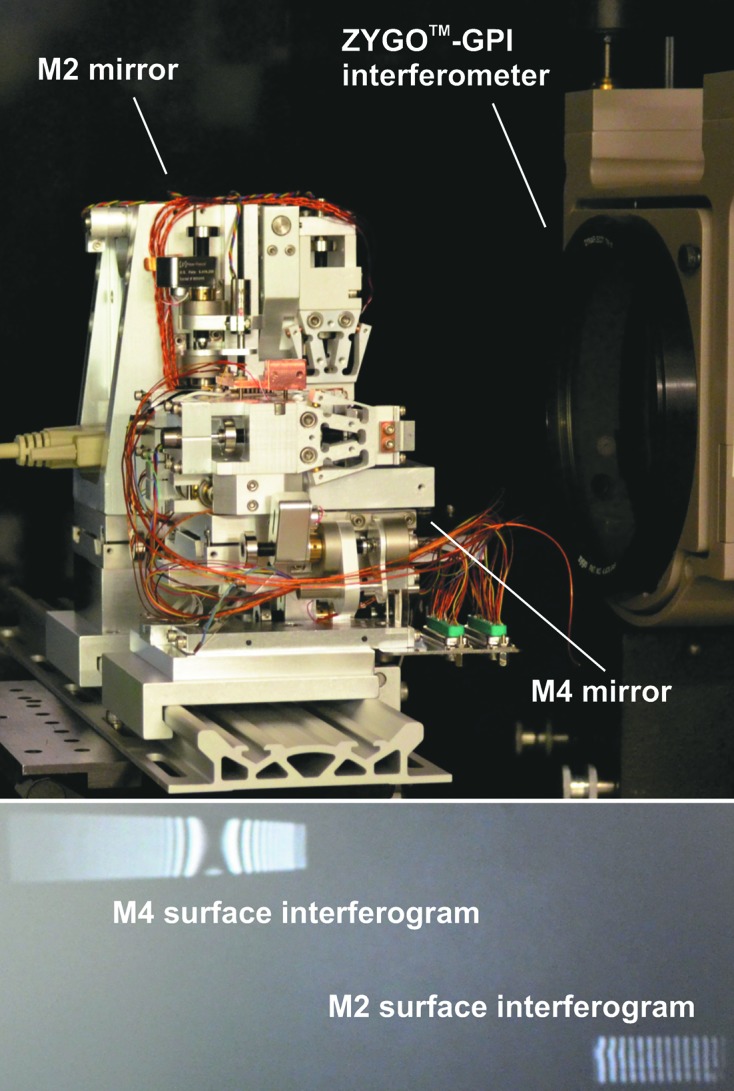
Experimental arrangement for roll angular alignment of the M2 and M4 mirrors. The ZYGO^TM^ GPI interferogram from the M4 mirror corresponds to a slight tangential tilt and absence of roll angular misalignment of the mirror with respect to the interferometer wavefront. The curved shape of the fringes is a signature of the anticlastic bending of the M4 mirror substrate. The M4 surface interferogram suggests perfect roll angular alignment but a tangential tilt of the mirror. Note that the M2 mirror fringes are significantly less curved due to the relatively smaller desired curvature, compared with that of the M4 mirror.

With all end-station mirrors assembled and pre-shaped, mutual roll alignment of the mirrors is performed by adjusting the roll angular tilts of the M2 and M4 mirrors. The normal-incidence surface interferogram of the vertically reflecting M3 mirror, recorded with the interferometer through a precision pentaprism, is used as a reference for roll alignment of the horizontally deflecting M4 mirror. A figure of merit for the alignment is a normal-incidence interferogram of the M4 surface, simultaneously recorded with the interferometer, without a noticeable roll tilt. Next, the roll alignment of the M2 mirror is performed in a similar manner using the M4 mirror as a reference. Fig. 5 ▶ illustrates the alignment procedure and reproduces the corresponding surface interferograms for the M2 and M4 mirrors. The accuracy of the described procedure is better than 0.1 mrad, which is good enough for BL 10.3.2. If necessary, a more accurate roll alignment can be achieved using a surface-slope profiler. In this case, we minimize the sagittal surface slope variation, measured with the LTP when scanning along the tangential direction.

## 4. Precision *ex situ* characterization and tuning of the mirror bending mechanism   

Optimal *ex situ* tuning of the mirror bending couples is performed with the upgraded long trace profiler LTP-II, available at the XROL (Kirschman *et al.*, 2008 ▶; McKinney *et al.*, 2010 ▶; Artemiev *et al.*, 2012 ▶). In order to obtain slope measurement accuracy on the level of 0.1 µrad using the LTP, we apply a number of experimental methods and procedures developed to suppress random noise due to air convection (Yashchuk *et al.*, 2006 ▶) as well as measurement errors, associated with instrumental temporal drift (Yashchuk, 2009 ▶) and systematic effects (Ali *et al.*, 2010 ▶; Yashchuk *et al.*, 2013*b* ▶). Each run of the slope profile measurement consists of a large number of scans (usually eight), which alternate in the scanning direction and arrangement of the mirror with respect to the LTP. Thus by averaging over eight LTP scans in the forward (F) and backward (B) directions performed in the order F-B-B-F-B-F-F-B, the temporal drift error, described by a third-order polynomial, is effectively suppressed (Yashchuk, 2009 ▶). Application of the drift suppression method allows starting LTP measurements practically without delay for temperature stabilization of the instrument itself. Nevertheless, when working with BL 10.3.2 end-station mirrors, a 1 h delay is necessary for the temperature of the mirror to be equilibrated after activation of the temperature stabilization system. In order to ensure the correspondence of the shape tuned in the laboratory to the one at the beamline, the temperature set point is adjusted to that of the beamline environment. With the active temperature stabilization deactivated, the surface shape of the assembled mirror is highly sensitive to temperature variation (Yuan *et al.*, 2010*a* ▶).

We follow the procedure described by McKinney *et al.* (2009 ▶, 2012 ▶) and Yashchuk *et al.* (2013*c* ▶) to optimally tune the bending couples using the LTP. The method assumes that the surface slope has an approximately linear response to changes of the bending couples:
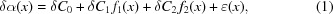
where 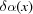 is the change of slope at position *x* on the mirror, resulting from an overall tilt 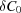, and changes 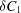 and 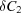 of the upstream and downstream bending couple actuator positions. The functions 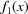 and 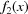 are referred to as the benders’ respective characteristic functions (McKinney *et al.*, 2009 ▶). The term 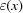 is the residual error of the linear model. The characteristic functions 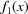 and 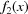 are estimated by applying successive known changes to each actuator and measuring the resulting slope difference, normalized to the changes. By performing a least-squares fit of the measured slope error profile to these characteristic functions, we determine the changes needed to minimize RMS slope deviation of the profile with respect to the desired shape.

**Figure 6:**
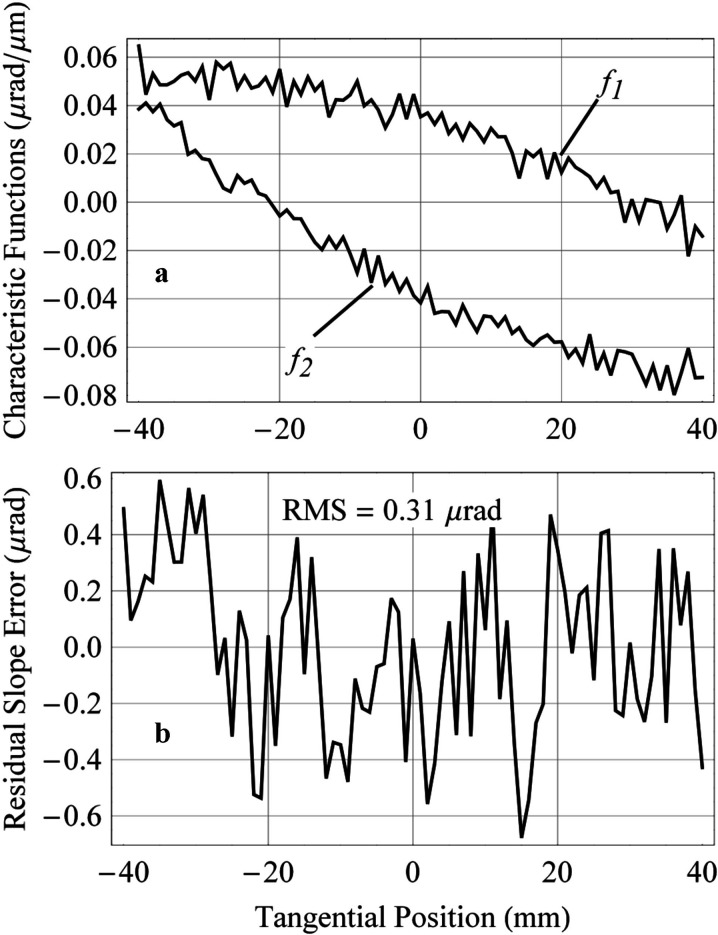
LTP measurements of (*a*) the characteristic functions 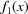 and 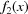 of the upstream and downstream bending couples, respectively, and (*b*) the residual (after subtraction the desired parabolic shape) surface slope error after tuning optimally.

The experimentally measured characteristic functions of the M2 benders are presented in Fig. 6(*a*) ▶. The slope profiler test with the mirror, pre-shaped with the interferometer, indicates a typical error of the pre-shaped figure of a few microradians. In order to compensate the error, we adjust the benders’ actuators by 50–100 µm. The total deflection of the cantilever springs is a few millimetres; and the resolution of the adjustment is better than 1 µm. After optimal adjustment of the M2 mirror shape, the RMS residual slope error is on the level of 0.3 µrad (Fig. 5*b* ▶), limited by the polishing quality.

## 5. At-wavelength fine tuning of the collimating mirror   

With the mirror assembled and the surface of the substrate precisely tuned to best fit the desired parabolic shape, the mirror M2 is ready for use at BL 10.3.2. However, the mirror application conditions inside the beamline end-station vacuum chamber are significantly different from that of the XROL, mainly due to the heat load from X-ray absorption. The actual optical geometry of the beamline can be noticeably different from the design. Hence, for truly optimal performance, the mirrors must be further tuned at the beamline using *in situ* metrology methods. At the ALS XROL, efficacy of at-wavelength metrology was demonstrated, in particular by reaching diffraction-limited focusing with a KB pair of mirrors with a design similar to the BL 10.3.2 end-station mirrors (Yuan *et al.*, 2010*b* ▶, 2011 ▶; Merthe *et al.*, 2011 ▶, 2013*a* ▶,*b* ▶).

Metrology techniques for optimal at-wavelength tuning of focusing bendable mirrors are well established [for a review, see, for example, Kewish *et al.* (2010 ▶), Goldberg *et al.* (2013 ▶), Fukui *et al.* (2013 ▶), Sawhney *et al.* (2013 ▶), Idir *et al.* (2013 ▶) and references therein]. The simplest and most prevailing is the scanning slit technique (Hignette *et al.*, 1997 ▶; Yashchuk *et al.*, 2013*c* ▶), where the focal plane ray error as a function of the transverse position of the slit is measured. The optimization algorithm is the same as the one for the *ex situ* tuning, discussed in §4. It utilizes the characteristic functions obtained by taking the difference of traces of the ray errors, arising from a unit change of the corresponding bending couple. Once the characteristic functions are measured, the optimal bending couples are determined by linear regression analysis of the measured ray error trace.

In this simplest realisation, the scanning slit method is not applicable to collimating mirrors, such as the BL 10.3.2 M2 mirror. Below we describe a modification of the method, which takes advantage of the monochromator placed downstream of the collimating mirror. The idea is to measure and minimize (by tuning the mirror shape) the variation of the energy of X-rays selected by the monochromator as a function of the tangential position along the mirror surface, or, equivalently, the transverse position of the slit placed between the mirror and monochromator. Such tuning can partially correct some imperfections of the monochromator as well.

Measurements of X-ray absorption spectra (XAS) on BL 10.3.2 are carried out by scanning the pitch angle of the monochromator. The second crystal is translated with respect to the first to maintain a fixed exit height. A slope error of 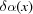 at some point *x* on the surface of M2 creates an angular ray error of 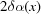 in the deflected ray. This angular error translates into an error 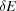 of the energy selected by the monochromator, given by
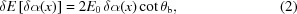
where 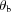 is the Bragg angle and 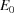 is the energy of X-rays, corresponding to 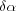 = 0. The combined effect of the energy errors is a broadening and, possibly, a displacement of the energy distribution of X-rays, transmitted at a given pitch angle of the monochromator. By scanning across the beam a narrow slit, placed between the M2 mirror and monochromator, one can isolate the contributions of energy error from different parts of the mirror and measure a trace of 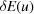, where *u* is the position of the slit in the transverse direction. The absolute value of 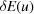 at a given *u* is determined as the monochromator pitch angle position corresponding to the absorption edge of a suitable absorber sample moved in the beam.

In the course of *in situ* optimization of the M2 shape, we scan the pitch angle of the monochromator such that the transmitted energy varied about the copper *K*-absorption edge (8980.45 eV). We chose Cu as a compromise between the increase in 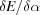 one obtains on going to higher energy, and the increasing natural widths of the absorption edges for heavier elements. Furthermore, the Cu edge has a distinctive feature (dip) which is not found on the edges of neighbouring elements. A thin copper foil is placed in the beam near the sample position, in order to obtain the spectrum. A PIN diode behind the copper foil measures the absorbed light, while a short-path ion chamber between M4 and the sample position provides the incident-beam normalization [see, for example, Marcus *et al.* (2004 ▶) and references therein]. In order to simplify the M2 alignment procedure, the mirror M3 was retracted from the beam.

**Figure 7:**
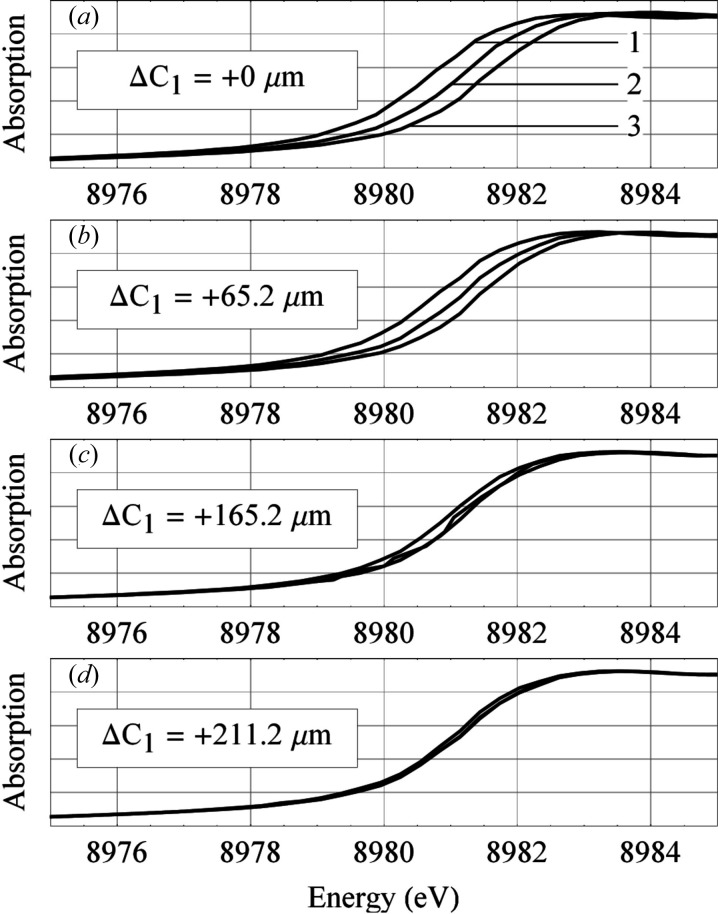
Alignment of M2 using the copper *K*-absorption edge. For the different positions 1, 2 and 3 of the scanning slit, (*a*) the absorption curves were initially separated, indicating poor collimation of the beam. By tuning the upstream bending couple, (*b* and *c*) the collimation was sequentially improved. At the optimal setting, (*d*) the absorption curves overlapped. The absorption scale is relative. Note that these curves extend only to the dip at the edge, not all the way up to the post-edge region.

Fig. 7(*a*) ▶ shows the absorption curves, 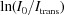, measured right after the mirror was placed on the beamline end-station. Each of the curves corresponds to one of three different positions of the scanning (JJ) slits, closed to a vertical width of 20 µm. At these positions, the downstream (1), middle (2) or upstream (3) end of M2 was illuminated. The differing slope errors of each illuminated portion of the mirror results in spectra that are displaced in energy. The pitch angle of the monochromator has been mapped to an energy scale, given by calibration. The offset of each curve was found by shifting curves 1 and 3 to find the best overlap with curve 2. The standard deviation offset of the initially measured absorption curves, shown in Fig. 7(*a*) ▶, was 0.46 eV.

**Figure 8:**
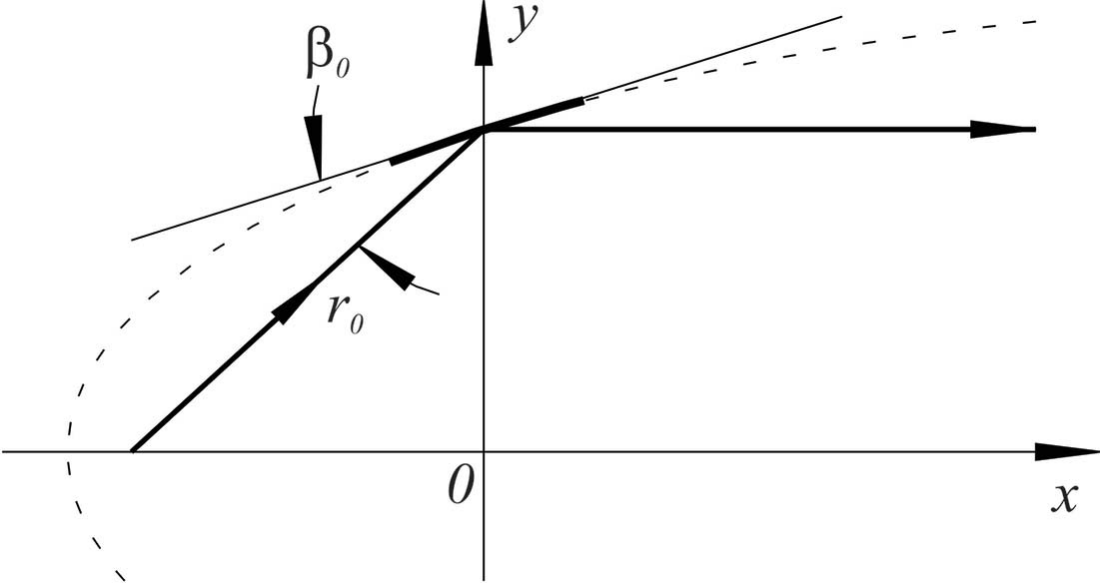
Parabolic cylinder mirror defined *via* the conjugate parameters: the grazing-incidence angle, 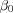, and the distance from the source to the mirror center, 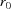.

In the case of a bendable parabolic cylinder mirror, there are generally three parameters that affect the mirror shape and, therefore, its collimating property. These are the mirror pitch (grazing incidence) angle 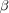 and two bending couples, 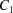 and 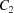. In our case, the collimating property of M2 is very sensitive to mirror pitch alignment. This can be understood from the difference of the surface slope functions of parabolas optimized for the same distance to the source, 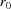, and slightly different grazing angles, 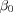 and 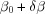. By differentiating by 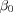 the surface slope function of a parabolic cylinder mirror (Fig. 8 ▶), expressed *via* the conjugate parameters 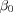 and 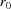 [note that the corresponding equation of McKinney *et al.* (2011 ▶) is inaccurate],
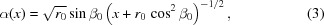
one can obtain the first-order approximation for the slope error due to the mirror pitch misalignment 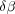: 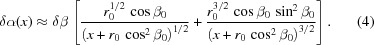
At the beamline design values of the conjugate parameters 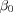 = 4.0 mrad and 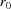 = 1.334 mm, the peak-to-valley (PV) variation of the slope error is
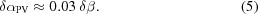
From equation (5), the PV error 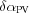 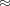 0.3 µrad, comparable with the r.m.s. residual slope error of the optimally shaped mirror (see Fig. 6 ▶), arises at 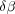 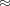 10 µrad. Besides the strong perturbation of the energy resolution, the change of the mirror pitch angle deflects the collimated beam by ∼2δβ, correspondingly shifting the overall energy range. Therefore, the mirror pitch angle should be accurately aligned before adjusting the mirror benders.

In order to analyse the effect of the bending couples to the monochromator resolution, we differentiate equation (3) by 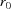 and derive the first-order approximation of the surface slope error due to the error in the couples, expressed as a source-to-mirror distance misalignment 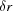:
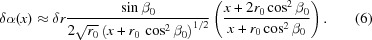
For the case of BL 10.3.2, the corresponding PV slope error is
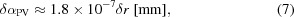
which gives 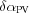 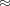 0.36 µrad at 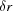 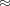 2 mm. Because the vertical width of the beamline roll slits is typically much larger than the diffraction limit size, the apparent uncertainty of the source (focus) position is practically even larger. The defocus effect leads to the observed large displacement of the absorption curves in Fig. 7(*a*) ▶.

The optimal source-to-mirror distance, 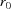, and the radius of curvature in the centre of the mirror, 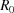, are connected through the focusing equation:
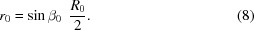
In order to express 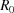 *via* the bending couples 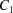 and 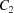, we use the Bernoulli–Euler equation (Ugural & Fenster, 1995 ▶), describing the bending of the mirror substrate with the overall length *L*,
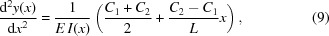
where *E* is the elastic modulus and 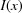 is the moment of inertia of the substrate. 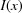 depends on the substrate’s sagittal width and the thickness that can generally be the functions of *x* [see, for example, McKinney *et al.* (2011 ▶) and references therein]. The variation of M2 substrate width is very small. We rewrite equation (9) in terms of the radius of curvature 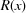, assuming 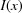 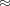 const = 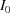,
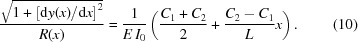
Equation (10) gives an approximation,
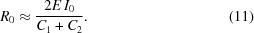
Finally, substituting (11) into (8), we obtain an approximate relation between the source-to-mirror distance and the bending couples:
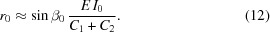
According to (12), the focal distance of the parabolic cylinder mirror can be optimized by aligning the mirror pitch (grazing incidence) angle and/or by tuning, at least, one of two bending couples.

The effect of the upstream bender adjustment to energy separation of the absorption curves, corresponding to different areas of the M2 mirror, is illustrated in Figs. 7(*b*)–7(*d*) ▶. With the optimally tuned upstream bender, we obtained the set of absorption curves (Fig. 7*d* ▶), where the standard deviation offset was 0.05 eV. This energy offset was at the level of uncertainty of the measured energy.

In addition to the suppression of the defocus error, discussed above, optimal adjustment of the two benders effectively compensates a coma-like error. To find the optimal bender settings, one applies the same technique based on linear regression, as discussed in §4. Let the value 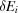 be the relative energy offset of the absorption curve for the *i*th slit positions, analogous to the numbering convention as in Fig. 7 ▶. Substituting (1) into (2), we see that the response of each 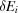 is linear in small changes of the bending couples:
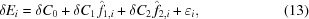
where 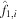 = 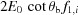 and 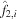 = 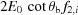 are the energy-based characteristic functions of the benders, and 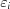 is the residual error of the model. In order to estimate the characteristic function, a change of the bender actuator position is applied and the normalized position difference of the resulting absorption curves is calculated. Obtaining in the linear regression analysis a confident prediction for optimal adjustments of 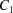 and 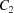 requires more than three JJ slit positions. In our case, nothing but an adjustment 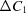 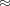 210 µm to the upstream bender was predicted with confidence. By performing this, we improved the vertical collimation of the beam incident on the monochromator tenfold and, correspondingly, decreased the M2-caused perturbation to the monochromator resolution by a factor of ∼10.

## 6. Conclusions   

ALS BL 10.3.2 is a multi-purpose microprobe for environmental and material science applications. The overall performance of this beamline, in terms of spatial and energy resolution, is sensitive to the alignment of its parabolic cylinder collimating mirror (M2) placed before the monochromator. Several major improvements were made to the design of this mirror, in order to enhance its beamline quality. We have presented details of the mirror bender design and *ex situ* metrology techniques, which allowed us to precisely assemble, align and shape the mirror, as well as to gain the mirror stability and extend the mirror operating lifetime by a factor of ∼10.

We have demonstrated the high efficacy of *in situ* alignment optimization of the M2 parabolic cylinder collimating mirror, using a newly developed method that employs the beamline’s monochromator. The method uses energy resolution as its figure of merit, which is a natural metric for the beamline performance. Application of the method to optimization of beamline performance of the M2 mirror enables us to reduce the collimation-induced energy spread from 0.46 eV to ∼0.05 eV.

This development of broadly applicable techniques and procedures is part of a broader effort to upgrade optics at the ALS and to establish highly accurate and transferable at-wavelength metrology methods.

## Footnotes

1 Macro Sensors Inc., CD 375 Series Miniature AC LVDT Position Sensors, http://www.macrosensors.com/CD_375.html.
